# Individualized Early Prediction of Familial Risk of Dyslexia: A Study of Infant Vocabulary Development

**DOI:** 10.3389/fpsyg.2017.00156

**Published:** 2017-02-21

**Authors:** Ao Chen, Frank Wijnen, Charlotte Koster, Hugo Schnack

**Affiliations:** ^1^Department of Psychiatry, Brain Center Rudolf Magnus, University Medical Center UtrechtUtrecht, Netherlands; ^2^Utrecht Institute of Linguistics OTS, Utrecht UniversityUtrecht, Netherlands; ^3^Center for Language and Cognition Groningen, University of GroningenGroningen, Netherlands

**Keywords:** dyslexia, vocabulary acquisition, machine learning, predictions, developmental trajectories

## Abstract

We examined early vocabulary development in children at familial risk (FR) of dyslexia and typically developing (TD) children between 17 and 35 months of age. We trained a support vector machine to classify TD and FR using these vocabulary data at the *individual* level. The Dutch version of the McArthur-Bates Communicative Development Inventory (Words and Sentences) (N-CDI) was used to measure vocabulary development. We analyzed group-level differences for both total vocabulary as well as lexical classes: common nouns, predicates, and closed class words. The generalizability of the classification model was tested using cross-validation. At the group level, for both total vocabulary and the composites, the difference between TD and FR was most pronounced at 19–20 months, with FRs having lower scores. For the individual prediction, highest cross-validation accuracy (68%) was obtained at 19–20 months, with sensitivity (correctly classified FR) being 70% and specificity (correctly classified TD) being 67%. There is a sensitive window in which the difference between FR and TD is most evident. Machine learning methods are promising techniques for separating FR and TD children at an early age, before they start reading.

## Introduction

Developmental dyslexia is an impairment of reading and spelling skills despite normal intellectual abilities and educational opportunities (Shaywitz and Shaywitz, 2005). The estimates of prevalence of dyslexia vary from 3 to 10%, depending on measures and inclusion criteria. There is wide agreement that dyslexia has a genetic basis (Francks et al., 2002; Galaburda et al., 2006; Schumacher et al., 2007). Even though a large proportion of children at family risk (FR) do not develop dyslexia, they still perform more poorly than typically developing (TD) children on tasks such as spelling, non-word reading, and reading comprehension (Pennington and Lefly, 2001; Snowling et al., 2003; Lyytinen et al., 2005). Various studies have demonstrated deviations in speech and language development in dyslexic and FR children prior to formal instruction in reading and writing (Koster et al., 2005; van Zuijen et al., 2012; van der Leij et al., 2013), but these markers have rarely been used to predict (the risk) of dyslexia on an individual level. The current study examines the vocabulary development of TD and FR infants from 17 to 35 months (Study I). Besides the group level comparison, we adopt a new approach, namely a machine learning technique, to predict the risk of dyslexia of *individual* infants and toddlers using their receptive and productive vocabularies (Study II). The present study is intended as a proof of concept, and the ultimate goal of our research is to predict the risk of dyslexia at an early age, as individual detection of a high risk of dyslexia at an early age will enable early preventative interventions, and may thus spare affected children an unfavorable start of their educational career.

### Links between Vocabulary Development and Reading Achievement

Impairment in phonemic awareness, that is, the ability to decompose words into constituent phonemes, has been claimed to be a core deficit in dyslexia (Swan and Goswami, 1997; Ramus, 2003; Goswami et al., 2011; Suk-Han Ho et al., 2011; Melby-Lervåg et al., 2012). It has been argued that poor phonemic awareness results in difficulty mapping phonemes onto graphemes. Phonological deficits in people with dyslexia persist into adulthood, even when visual word recognition difficulty has often been compensated (Bruck, 1992; Wilson and Lesaux, 2001).

Vocabulary and phonological ability have been hypothesized to mutually influence each other. On the one hand, learning native phonological contrasts can facilitate vocabulary development (Werker and Curtin, 2005; Swingley, 2009). Knowledge of native phonemes may help infants learn words (Swingley, 2009). On the other hand, vocabulary expansion has been proposed as a driving force for segmenting words into phonemes, which is a prerequisite for phonological awareness to emerge. The *lexical restructuring model* (LRM) hypothesizes that words are represented holistically at the initial stage of word learning. As infants’ vocabulary expands, comparison between lexical entries allows children to decompose words into increasingly smaller units, and ultimately children become aware of phonemes being the constituents of words. Recognition of phonemes provides the basis for phoneme-grapheme mapping, the backbone of reading (Metsala and Walley, 1998; Walley et al., 2003). Seeing the mutual influence between phoneme learning and word knowledge, it is expected that the deeply rooted phonological awareness impairment is reflected in vocabulary acquisition. Indeed, correlations between preschool vocabulary and phonological awareness as well as later reading abilities have been found in multiple studies (Chaney, 1998; Olofsson and Niedersøe, 1999; Cooper et al., 2002; Kim et al., 2014). Dyslexic as well as FR children have been found to differ from TD children in terms of vocabulary before formal reading instruction starts (Scarborough, 1990; Snowling et al., 2003; Fowlert et al., 2004; Koster et al., 2005; Lyytinen et al., 2005; Carroll et al., 2014).

Several studies have attempted to identify precursors of dyslexia from children’s vocabulary before they start reading. Scarborough (1990) compared language abilities of FR and TD children at age 2.5, 3, and 5. At age 3, those FR children who later developed reading difficulties had smaller receptive vocabularies. Snowling et al. (2003) showed that at 3 years and 9 months, FR children who eventually became manifestly dyslexic had smaller receptive as well as productive vocabularies than the TD children.

Lyytinen and Lyytinen (2004) and Koster et al. (2005) used the MacArthur–Bates Communicative Development Inventories (CDI; Fenson et al., 1993) to examine whether TDs and FRs’ vocabulary differed at a younger age. The Dutch Dyslexia Programme (DDP) examined TD and FR children’s language development longitudinally from 2 months to 9 years (van der Leij et al., 2013). In DDP, Koster et al. (2005) compared the vocabulary composition of 17-month-old TD and FR infants using the Dutch version of the CDI. The TD infants on average had larger productive vocabularies than the FR ones (34.7 vs. 27.5). In addition, the FR and TD infants showed different vocabulary profiles. Among those infants who produced more than 50 words in total, the TDs produced more verbs and closed-class words than the FRs; such a difference was not observed for those who produced less than 50 words. Lyytinen and Lyytinen (2004) studied Finnish-learning TD and FR children at ages 2, 2.5, 3.5, and 5 years. When the children were two years old, their parents filled in the Finnish version of the CDI. At this age, no significant group difference was found for total CDI production scores, but at the later ages, the FR children showed a smaller productive vocabulary when tested with a different task (Boston Naming Task: BNT). Lyytinen and Lyytinen (2004) and Koster et al. (2005) yielded partly inconsistent results regarding the ages at which TD and FR differ in vocabulary, which may be due to infants’ different language backgrounds.

In light of these results, we ask whether TD and FR children exhibit a different vocabulary developmental trajectory, and as a proof of concept, whether early vocabulary measures can predict familial risk (FR) at the individual level. After an initial period of relatively slow growth, children’s vocabularies rapidly increase starting around 15 months (Bates et al., 1995). If phonology and vocabulary mutually enhance each other, and seeing the deeply rooted phonological difficulties of dyslexics, it is reasonable to hypothesize that infants who will develop dyslexia in the end may differ from TD children when going through this vocabulary spurt. So far however, TDs’ and FRs’ early vocabulary development profile has never been captured. The studies on vocabulary development as related to dyslexia examined children who either already passed the vocabulary spurt period (Scarborough, 1990; Snowling et al., 2003), or tested ages with long time lag in between (Lyytinen and Lyytinen, 2004). In addition, for children of different ages, different testing instruments were used, which could be a confounding factor for the results. To fill in the voids, the present study examined the vocabulary of Dutch TD and FR children at 17, 18, 19, 20, 23, 29, and 35 months, using the Dutch version of the CDI (N-CDI; Zink and Lejaegere, 2002) which zoomed into the vocabulary spurt stage and ensured the validity of cross-age comparison.

### Individual Prediction Using Machine Learning

In previous studies, logistic regression models have been used to predict dyslexia (Puolakanaho et al., 2007; Thompson et al., 2015). In such models, multiple *a priori* determined language ability measures, together with the status of FR, have been used as predictors. However, these studies are largely *correlational* rather than *predictive*, as they did not test cases outside the sample that was used to build the model. Hence, a true predictive model is still missing.

Machine learning is a widely used pattern recognition technique for making quantitative predictions. Algorithms are trained to discover regularities in the input data that are related to the quantity of interest without any predetermined factors. In such a bottom-up fashion, machine learning may find discriminative features between groups which are not considered *a priori*. Machine learning has been successfully applied in multiple areas, such as classification of different psychiatric disorders (Schnack et al., 2014) and separation of control infants and infants at-risk of autism (Bosl et al., 2011).

To gain knowledge on whether the risk of dyslexia can be predicted at an individual level, in the current study we try to predict whether each individual child is FR or TD by applying a machine learning tool. In our case, the machine learning algorithm has to find a pattern in the N-CDI scores that predicts whether an infant is FR or TD. The generalizability of the resulting prediction model is tested by applying it to new cases. Because of the relatively small sample size per age group, we choose to train linear prediction models, to avoid overfitting. We used the linear Support Vector Machine (SVM), a high-dimensional pattern recognition algorithm (Vapnik, 1999). SVM does not only classify the cases, but also indicates in a straightforward way which features contribute to the classification and to what extent. It should be noted that other linear algorithms, such as logistic regression, linear discriminative analysis, and lasso could yield comparable results (Janousova et al., 2016; Kassraian-Fard et al., 2016).

### Aim of the Study

The aim of the current study is two-fold. The first is to examine the trajectory of early vocabulary development of FR and TD toddlers using one single instrument, namely the Dutch version N-CDI. We specifically looked into the period during which infants go through rapid vocabulary increase (17–35 months). If, as assumed, phonological ability and vocabulary growth mutually influence each other (Metsala and Walley, 1998; Werker and Curtin, 2005), seeing the deeply rooted phonological deficits of dyslexics, the FR children should show a different vocabulary developmental trajectory compared to TD children. Our second aim is to use the SVM algorithm to predict whether an individual child is FR or TD, as a proof of concept. To our knowledge, this is the first effort in *predicting* the risk of dyslexia at an *individual* level, where the child whose risk is to be predicted is not part of the sample from which the prediction model is constructed. This study is also the first one to predict the at-risk status at such early age, and we make use of machine learning as a proof of concept. It should be acknowledged that not all FR infants will develop dyslexia, but they have a higher chance to develop dyslexia than the TDs, and those who do not develop dyslexia still perform moor poorly than TDs in reading (Pennington and Lefly, 2001; Snowling et al., 2003; Lyytinen et al., 2005; Shaywitz and Shaywitz, 2005). Hence, it is expected that the FRs exhibit the characteristics of the dyslexics, and can be discriminated from TDs at individual level. We will follow these children up and optimize the prediction model once the final reading status of the children are known.

## Materials and Methods

### Ethics Statement

As the study was non-invasive, it did not require ethic approval. Informed consent was obtained from parents of all the participants.

### Participants

Two independent samples, taken from the Dutch Dyslexia Program (DPP) and Utrecht Dyslexia-Language Impairment Study (UDySLI), respectively, were used in the current study. Only the data of monolingual Dutch children were used. We included 476 children in total. Four age groups, viz. the 17-, 23-, 29-, and 35-month-olds (all within two weeks of each age, e.g., 17:01–17:14) were taken from the DDP cohort. The 17-month-old infants included in the current study partly overlap with those analyzed in Koster et al. (2005). Another three age groups, viz., the 18-, 19-, and 20-month-olds (all within one month of each age, e.g., 18:01–18:29) were from UDySLI. The DDP children often had repeated measurements, whereas the UDysSLI children had single measurement moments. For 251 children, N-CDI scores were obtained once; 43 children were scored at two different ages, 72 were measured at three different ages, and 110 were measured at four different ages, resulting in a total of 993 N-CDI score sheets. The children were labeled as FR if at least one of the parents was reading impaired^1^, which was determined by three tests administered at either the Utrecht or Groningen labs. Two of them were reading tests, namely the ‘Een-Minuut-Test’ (EMT; Brus and Voeten, 1973), and the ‘Klepel’ (van den Bos et al., 1994). The other test was the comprehension subscale of Wechsler Adult Intelligence Scale (WAIS; Wechsler, 1997). A parent was reading impaired if he/she had a score at the lowest 10% in one of two reading tasks, or at the lowest 25% in both, or he/she had a discrepancy larger than 60% between a high score on the WAIS comprehension subscale and the score on one of the reading test. **Table 1** lists the number of included TD and FR participants in each age group. As there were much fewer children in the 19- and 20-month-old subgroups than in the other age groups, these two were collapsed to form the 19–20-month-old group for the purposes of statistical analysis and machine learning.

**Table 1:** Numbers of boys and girls in the typically developing (TD) and familial risk (FR) groups at different ages.

Age	TD girls	TD boys	FR girls	FR boys	Total
17m	47	53	47	52	199
18m	28	29	28	29	114
19m	9	20	9	20	58
20m	16	15	16	15	62
23m	48	54	47	56	205
29m	40	55	40	54	199
35m	38	46	38	44	166

### Materials and Design

All parents were asked to fill in the Dutch version of the CDI (N-CDI) on paper. On the basis of the parents’ report, two sets of measurements were calculated: (1) total vocabulary; (2) composite classes, adopted from Koster et al. (2005; Caselli et al., 1995). From the 22 word categories listed in N-CDI, three major composite word classes were constructed, namely common nouns, predicates, and closed class words. The N-CDI categories included in each composite are listed in **Table 2**. The remaining categories that cannot be grouped into any composite were grouped in the composite class “other^2^”. For each word listed in the CDI, parents were asked to choose between “understands but does not produce yet” and “understands and produces”. For the total vocabulary, the composites, and each individual category, a receptive score which equaled the sum of all checked words, and a productive score which equaled the sum of all words checked as “understand and produce”, were calculated.

**Table 2:** Word examples in each N-CDI category, separated by composites.

Common nouns	Predicates	Closed class words	Other
Animals (e.g., monkey *aap*)	Action words (verbs) (e.g., stay *blijven*)	Pronouns (e.g., I *ik*)	Sound effects (e.g., meow *miauw*)
Vehicles (e.g., car *auto*)	Descriptive words (adjectives) (e.g., night *nacht*)	Question words (e.g., how *hoe*)	Items outside the house (e.g., tree *boom*)
Toys (e.g., ball *bal*)		Prepositions and locations (e.g., above *boven*)	Places outside the house (e.g., bakery *bakker*)
Food and drink (e.g., patato *aardappel*)		Quantifiers and articles (e.g., all *alles/allemaal*)	People (e.g., baby *baby*)
Clothing (e.g., jacket *jas*)		Helping verbs (e.g., do *doen*)	Games and routines (e.g., brush teeth *tandjes poesten*)
Body parts (e.g., arm *arm*)		Connecting words (e.g., then *dan*)	Words about time (e.g., day *dag*)
Small household items (e.g., bord *plate*)			
Furniture and rooms (e.g., door *deur*)			

*The Dutch words are given in italics*.

We first examined the developmental trajectory by comparing TD and FR groups’ receptive as well as productive scores for both the total vocabulary and the composites at group level using MANOVA (Study I). As some children were scored several times, and some others only once, and since DDP and UDySLI recruited different children, it was not possible to collapse all the children into one statistical model and to use age as a factor. Therefore, separate MANOVAs were carried out for the receptive and productive scores for each age group separately. In the second part of our investigation (Study II), a SVM algorithm was trained with the N-CDI scores so as to predict whether an individual infant was FR or TD, using the composite scores and the scores of the individual categories, respectively.

### Study I: Developmental Patterns

#### Results

**FIGURE 1:**
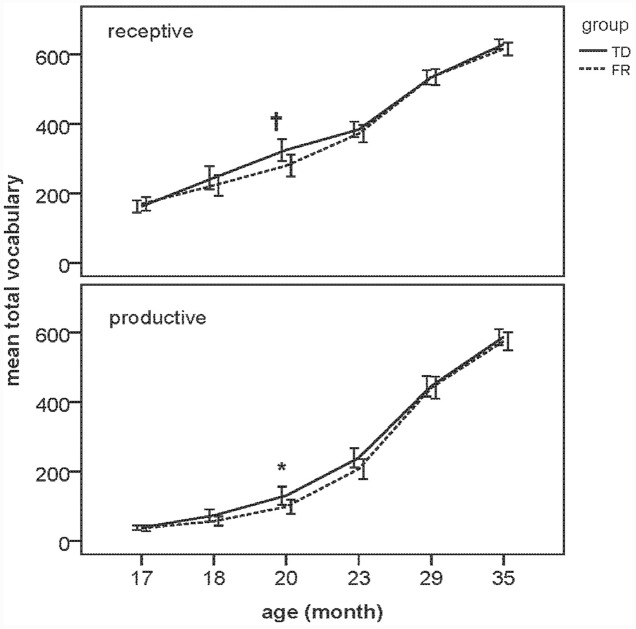
**Mean total receptive (TOTREC) and mean total productive vocabularies (TOTPRO) vocabulary of typically developing (TD) and familial risk (FR) at all ages**. ^∗^ indicates significant group difference (*p* < 0.05), and † indicates marginal significance (0.05 < *p* < 0.08). Error bars represent ± 2 SE.

With regard to total receptive (TOTREC) and total productive vocabularies (TOTPRO), a MANOVA was carried out with group (TD vs. FR) as the independent variable. A significant effect of group on TOTPRO [*F*(1,118) = 4.14, *p* < 0.05, $\eta_{p}^{2}$ = 0.034] was found in the 19–20 month olds only, indicating that productive vocabularies in the FR group were smaller than those in the TD group at this age. A marginally significant effect in the same direction was found for TOTREC in the same age group, *F*(1,118) = 3.83, *p* = 0.053, $\eta_{p}^{2}$ = 0.031. No significant group differences were found for either TOTREC or TOTPRO in any of the other age groups. Results of the MANOVAs are listed in **Table 3**. **Figure 1** shows the TOTREC and TOTPRO scores of all FR and TD children as a function of age. The total vocabularies of both TDs and FRs increased as they grew older. FRs had smaller vocabularies than TDs, yet only at 19–20 months.

**Table 3:** Mean total receptive (TOTREC) and productive scores (TOTPRO) of TD and FR.

		Mean TD (*SD*)	Mean FR (*SD*)	Comparison	*p*-value
17 m	TOTREC	163 (90)	170 (98)	*F*(1,197) = 0.29	0.65
	TOTPRO	38 (32)	36 (38)	*F*(1,197) = 0.20	0.59
18 m	TOTREC	245 (124)	223 (112)	*F*(1,112) = 0.96	0.33
	TOTPRO	73 (69)	57 (50)	*F*(1,112) = 2.14	0.15
19–20 m	TOTREC^∗^	326 (122)	280 (123)	*F*(1,118) = 4.14	0.04, $\eta_{p}^{2}$ = 0.034
	TOTPRO†	130 (101)	98 (77)	*F*(1,118) = 3.83	0.053, $\eta_{p}^{2}$ = 0.031
23 m	TOTREC	384 (113)	372 (140)	*F*(1,203) = 0.49	0.48
	TOTPRO	239 (140)	207 (145)	*F*(1,203) = 2.57	0.11
29 m	TOTREC	534 (102)	535 (110)	*F*(1,187) = 0.04	0.95
	TOTPRO	445 (141)	441 (154)	*F*(1,187) = 0.41	0.84
35 m	TOTREC	629 (65)	616 (86)	*F*(1,164) = 1.26	0.26
	TOTPRO	587 (105)	575 (116)	*F*(1,164) = 0.51	0.48

*^∗^Indicates significance at 0.05 level and ^†^indicates marginal significance (0.05 < *p* < 0.08 in all cases) between TD and FR as revealed by a MANOVA*.

**FIGURE 2:**
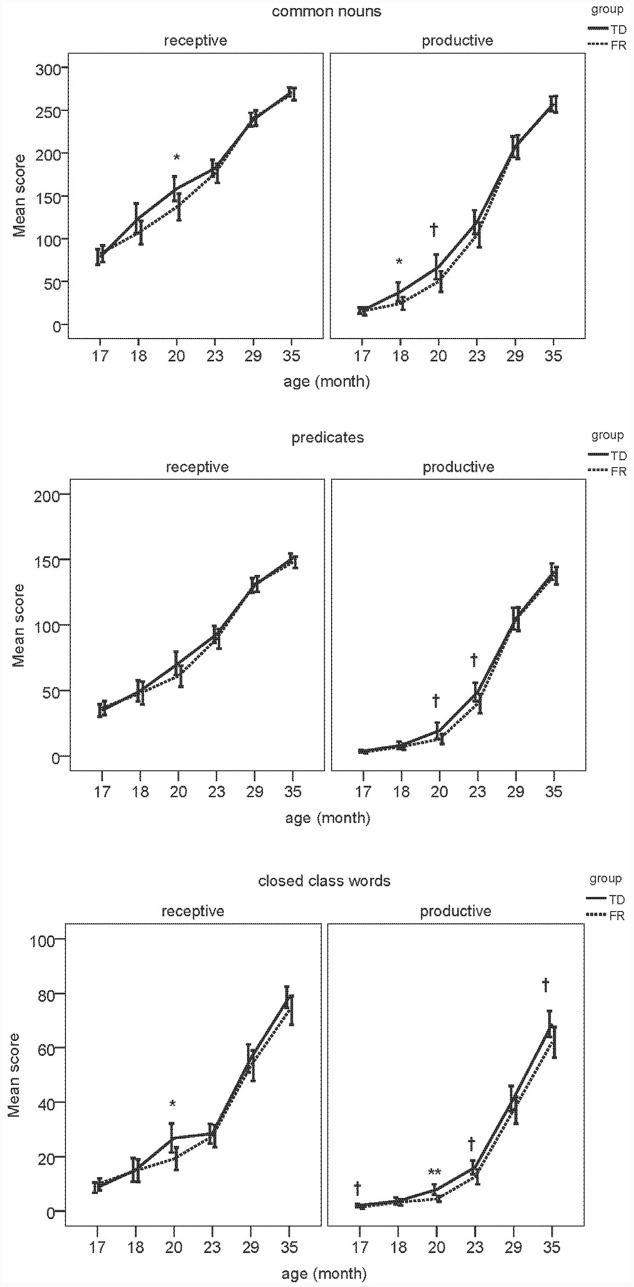
**Developmental trajectories of TD and FR of each composite**. Receptive and productive scores are depicted separately, where ^∗∗^indicates significant difference (*p* < 0.01), ^∗^indicates significant difference (*p* < 0.05), and ^†^indicates marginal significant (0.05 < *p* < 0.08) difference between TD and FR. Error bars represent ± 2 SE.

Next, we examined composite class scores of TD and FR children at each age. The results of the MANOVAs are presented in **Tables 4**–**9**. **Figure 2** shows the receptive (i.e., understanding only; REC) and productive (i.e., understanding and producing; PRO) composite sizes as a function of age, with significant group-level differences indicated. Similar to the total vocabularies, for the composites, the difference between TDs and FRs was most evident at 19–20 months, with FRs having a lower score than TDs.

**Table 4:** Typically developing and familial risk’s composite scores at 17 months.

17-month-olds	Mean TD (*SD*)	Mean FR (*SD*)	Comparison	*p*-value
Common nouns REC	78.7 (45.5)	82.4 (48.2)	*F*(1,197) = 0.31	0.58
Common nouns PRO	16.1 (17.7)	15.4 (23.0)	*F*(1,197) = 0.06	0.81
Predicates REC	35.0 (23.6)	36.7 (26.5)	*F*(1,197) = 0.24	0.62
Predicates PRO	3.9 (4.5)	3.0 (4.4)	*F*(1,197) = 1.86	0.17
Closed-class words REC	8.6 (9.4)	9.82 (11.0)	*F*(1,197) = 0.71	0.40
Closed-class words PRO^†^	2.11 (2.7)	1.46 (2.4)	*F*(1,197) = 3.17	0.077, $\eta_{p}^{2}$ = 0.016

*^∗∗^indicates significance at 0.01 level, ^∗^indicates significance at 0.05 level, ^†^indicates marginal significance (0.05 < *p* < 0.08), all *p*-values are Bonferroni adjusted. REC stands for receptive and PRO stands for productive*.

**Table 5:** Typically developing and familial risk’s composite scores at 18 months.

18-month-olds	Mean TD (*SD*)	Mean FR (*SD*)	comparison	*p*-value
Common nouns REC	123.8 (66.2)	107.0 (51.6)	*F*(1,112) = 2.27	0.14
Common nouns PRO^∗^	38.0 (42.2)	24.3 (27.8)	*F*(1,112) = 4.15	0.044, $\eta_{p}^{2}$ = 0.036
Predicates REC	49.8 (30.3)	48.1 (32.9)	*F*(1,112) = 0.09	0.77
Predicates PRO	8.3 (10.3)	7.1 (9.1)	*F*(1,112) = 0.38	0.54
Closed-class words REC	15.1 (16.4)	14.8 (15.5)	*F*(1,112) = 0.01	0.92
Closed-class words PRO	3.7 (4.7)	3.2 (4.5)	*F*(1,112) = 0.28	0.60

*^∗∗^indicates significance at 0.01 level, ^∗^indicates significance at 0.05 level, ^†^indicates marginal significance (0.05 < *p* < 0.08), all p values are Bonferroni adjusted. REC stands for receptive and PRO stands for productive*.

**Table 6:** Typically developing and familial risk’s composite scores at 20 months.

19–20-month-olds	Mean TD (*SD*)	Mean FR (*SD*)	Comparison	*p*-value
Common nouns REC^∗^	158.5 (55.0)	136.9 (60.6)	*F*(1,118) = 4.17	0.043, $\eta_{p}^{2}$ = 0.034
Common nouns PRO^†^	67.2 (55.9)	50.1 (47.0)	*F*(1,118) = 3.27	0.07, $\eta_{p}^{2}$ = 0.027
Predicates REC	70.8 (34.3)	61.1 (31.1)	*F*(1,118) = 2.65	0.11
Predicates PRO^†^	19.5 (23.4)	13.0 (14.4)	*F*(1,118) = 3.41	0.07, $\eta_{p}^{2}$ = 0.028
Closed-class words REC^∗^	26.8 (20.7)	19.2 (16.1)	*F*(1,118) = 5.07	0.026, $\eta_{p}^{2}$ = 0.041
Closed-class words PRO^∗∗^	7.9 (7.4)	4.5 (3.7)	*F*(1,118) = 10.50	0.002, $\eta_{p}^{2}$ = 0.082

*^∗∗^indicates significance at 0.01 level, ^∗^indicates significance at 0.05 level, ^†^indicates marginal significance (0.05 < *p* < 0.08), all *p*-values are Bonferroni adjusted. REC stands for receptive and PRO stands for productive*.

**Table 7:** Typically developing and familial risk’s composite scores at 23 months.

23-month-old	Mean TD (*SD*)	Mean FR (*SD*)	Comparison	*p*-value
Common nouns REC	182.3 (50.2)	176.4 (57.4)	*F*(1,203) = 0.61	0.44
Common nouns PRO	119.2 (70.9)	104.4 (73.4)	*F*(1,203) = 2.17	0.14
Predicates REC	93.0 (32.0)	89.5 (37.5)	*F*(1,203) = 0.50	0.48
Predicates PRO^†^	49.0 (34.9)	40.1 (36.8)	*F*(1,203) = 3.15	0.078, $\eta_{p}^{2}$ = 0.015
Closed-class words REC	28.4 (18.1)	27.5 (20.6)	*F*(1,203) = 0.11	0.74
Closed-class words PRO^†^	16.0 (13.0)	12.6 (13.7)	*F*(1,203) = 3.52	0.062, $\eta_{p}^{2}$ = 0.017

*^∗∗^indicates significance at 0.01 level, ^∗^indicates significance at 0.05 level, ^†^indicates marginal significance (0.05 < *p* < 0.08), all *p*-values are Bonferroni adjusted. REC stands for receptive and PRO stands for productive*.

**Table 8:** Typically developing and familial risk’s composite scores at 29 months.

29-month-old	Mean TD (*SD*)	Mean FR (*SD*)	Comparison	*p*-value
Common nouns REC	238.9 (39.0)	240.7 (43.4)	*F*(1,187) = 0.09	0.76
Common nouns PRO	207.4 (58.1)	207.1 (66.5)	*F*(1,187) = 0.00	0.98
Predicates REC	130.3 (26.6)	131.4 (28.5)	*F*(1,187) = 0.07	0.79
Predicates PRO	104.9 (40.5)	104.5 (43.2)	*F*(1,187) = 0.00	0.95
Closed-class words REC	56.1 (25.0)	53.4 (27.2)	*F*(1,187) = 0.55	0.47
Closed-class words PRO	41.1 (22.5)	37.0 (23.8)	*F*(1,187) = 1.62	0.21

*^∗∗^indicates significance at 0.01 level, ^∗^indicates significance at 0.05 level, ^†^indicates marginal significance (0.05 < *p* < 0.08), all *p*-values are Bonferroni adjusted. REC stands for receptive and PRO stands for productive*.

**Table 9:** Typically developing and familial risk’s composite scores at 35 months.

35-month-old	Mean TD (*SD*)	Mean FR (*SD*)	Comparison	*p*-value
Common nouns REC	271.3 (25.0)	268.7 (32.4)	*F*(1,164) = 0.35	0.56
Common nouns PRO	257.6 (38.9)	257.0 (43.7)	*F*(1,164) = 0.01	0.93
Predicates REC	151.1 (15.3)	147.9 (19.7)	*F*(1,164) = 1.40	0.24
Predicates PRO	140.8 (28.6)	137.7 (30.6)	*F*(1,164) = 0.45	0.50
Closed-class words REC	78.6 (17.8)	73.7 (24.0)	*F*(1,164) = 2.19	0.14
Closed-class words PRO^†^	68.7 (21.8)	62.0 (25.2)	*F*(1,164) = 3.38	0.068, $\eta_{p}^{2}$ = 0.02

*^∗∗^indicates significance at 0.01 level, ^∗^indicates significance at 0.05 level, ^†^indicates marginal significance (0.05 < *p* < 0.08), all *p*-values are Bonferroni adjusted. REC stands for receptive and PRO stands for productive*.

#### Discussion

The group-level difference between TD and FR children showed a consistent pattern for both the total vocabulary and composites. The FR children were surpassed by TD children in vocabulary development, but only within a restricted age window, namely around 20 months. At this age, the TD children had (marginally) significantly higher scores for both the separate composite classes (except receptive predicates) as well as total vocabulary. For both younger and older groups, group-differences are absent. These findings are consistent with Koster et al. (2005), in that vocabulary size appears to matter for distinguishing between TD and FR children. In Koster et al. (2005), once the children had a large enough productive vocabulary, i.e., between 50 and 100 words, TD children produced more verbs and closed-class words than FR children. As Koster et al. (2005) examined 17-month-old infants, most of them still had a very small vocabulary, and among a total of 192 infants, only 37 of them met the 50-word criterion. This casts doubt on the generalizability of the group level difference at this young age. In the current study, the 20-month-olds produced 114 words on average, close to the critical size proposed in Koster et al. (2005). Solely based on infants’ age, without applying an additional selection criterion derived from the size of the productive vocabulary, the difference between TD and FR is readily visible at 20 months. Indeed, the 17-month-old high producers were comparable to 20-month-old average producers in terms of vocabulary size. We also found an overall, rather than a “category specific”, disadvantage of FR at 20 months. The FR children not only lagged behind for predicates and closed-class words, but also common nouns. TDs and FRs may have different developmental patterns of brain structure and function, which may underlie the differences observed in vocabulary development (Leppänen et al., 2002; van Zuijen et al., 2012). This overall delay may relate to deficient phonological and/or general auditory abilities. Hence, the age window of 19–20 months may be the critical window for detecting vocabulary-related precursors to dyslexia. After 20 months, it seems that the vocabulary difference between TDs and FRs became weaker. The reduced difference might be due to two factors: first, the FR infants may have a delay in early vocabulary development. In other words, it takes FR more time to learn words than TDs. Such a delay was not visible for the 17-month-olds as at this early age, both TDs and FRs had very small vocabularies, which made the difference between the groups difficult to discern. Second, the CDI has a finite number of words, hence it is possible that after 20 months, the TDs know more words outside the CDI, which cannot be captured by the instrument used in the current study.

The closed class words seem to be particularly difficult for the FRs, especially in production, who lag behind the TDs at all the ages (although statistically non-significant at 18 and 29 months). For common nouns and predicates, for the age period tested, it seems that although FRs fall behind at 19–20 months, they catch up later. In contrast, the FRs’ difficulty with closed-class words seems to be longer lasting, and by 35 months, the FRs still had a lower score than the TDs. Closed class words seem to be difficult for children in general. Compared to common nouns and predicates, children acquire closed class words later (Bates et al., 1994; Goodman et al., 2008). Unlike content words, closed class words tend to be short and unstressed in speech, and often without a direct reference. A possible phonological and auditory deficit might particularly hinder FRs’ representation of the acoustically non-prominent closed class words, even when their difficulty with learning the salient content words has been compensated. In other words, FR fall behind when learning the more difficult words.

Vocabulary and phoneme knowledge are interdependent. Accurate representation of phonemes is a prerequisite for learning words, and distinguishing similar sounding words enhances phoneme representation in return (Metsala and Walley, 1998; Walley et al., 2003; Werker and Curtin, 2005). Seeing the deeply rooted phoneme awareness difficulties among dyslexics, it is likely that children who develop dyslexia later go through atypical vocabulary development. Our results indicate that indeed, the FR children were hindered at the initial stage of word learning. Such a delay may reflect and be due to impaired phonological ability. Lyytinen and Lyytinen (2004) tested Finnish TD and FR children’s vocabulary with CDI, and did not find group difference at age 2. Their finding is consistent with ours. It seems that although there is an initial delay, the FR children quickly catch up. Such a quick recovery suggest that they may develop compensatory strategies to fulfill the need for word learning. In other words, they may find alternative pathways for vocabulary development. For now, the children included in this study are not old enough to know the final reading status, it would be interesting to see in the future whether the lower scores of the FRs were driven by those who develop dyslexia in the end.

### Study II: Prediction at Individual Level Using Machine Learning

In the second part of our study, we employ the linear SVM, a supervised learning algorithm, to predict whether individual children are either FR or TD. The SVM classification process consists of two phases. In the first step, the SVM is provided with a labeled (TD, FR) training dataset, consisting of a set of properties, or features (N-CDI scores), for each case to be predicted. A model is then created from the training set, with the goal to find an optimal classification of the two groups based on their features. In the second phase this prediction model is validated in new, unseen, cases.

#### Step 1

Each participant *i* was labeled *t_i_* = –1 (TD) or *t_i_* = +1 (FR) and was represented by his/her features, the N-CDI scores, *x*_1_,.., *x_d_*, congregated into a *d*-dimensional vector ***x****_i_*. The SVM algorithm searches for a linear combination of these features that best predicts the each subject’s class. It tries to find the optimal set of weights *w*_1_,.., *w_d_*, and a bias (offset) *b*, so that the function *f*(***x****_i_*) = *w*_1_*x*_1_+..+*w_d_x_d_* –*b* < 0 if *t_i_* = –1 and *f*(***x****_i_*) > 0 if *t_i_* = +1. The resulting weights indicate the relevance of the features for the prediction. The sign of a particular feature’s weight indicates whether an increase (positive) or decrease (negative) of the feature’s value contributes to being classified as FR; furthermore, a larger absolute value of a feature’s weight reflects a more important role of the feature.

**FIGURE 3:**
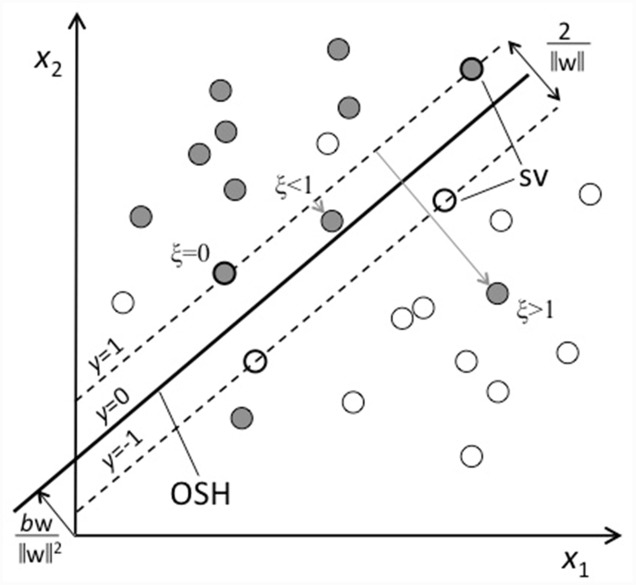
**Principles of support vector machine (SVM) classification**. The subjects from two groups, or classes, are represented by their feature vector *x*, i.e., their locations in a two-dimensional feature space according to their scores on features *x*_1_ and *x*_2_. Open circles represent one class and are labeled by ‘–1’ (e.g., typically developing, TD) and closed circles represent the other class and are labeled by ‘+1’ (e.g., at family risk). The classifier is trained to separate the two classes. The optimal separation is achieved when the space between the two classes, i.e., the margin, is as large as possible, and this separating hyperplane is called the optimal separating hyperplane (OSH; thick line). It is defined by *y* = *w*^T^*x*-*b* = 0, where *w* is the weight vector and *b* is an offset. The size of the margin is 2/||w||, so minimizing ||w|| maximizes the margin (indicated by the dashed lines, which are ‘supported’ by a subset of the subjects, the so-called support vectors (SV; thick circles)). Subjects will be allowed to lie on the wrong side of the OSH, but with a penalty depending on their distance to the OSH. Slack variables ξ are introduced to quantify this so-called soft margin: A correctly classified subject has ξ = 0, otherwise ξ is the distance between the OSH and the subject’s feature vector *x*.

Mathematically, these vectors ***x****_i_* exist in a high-dimensional space (a two-dimensional example is shown in **Figure 3**). The algorithm is designed to create an optimal separation between the two classes by constructing a flat decision surface (hyperplane) in such a way that the space between the two classes, i.e., the margin, is as large as possible. The larger the margin is, the better the classifier’s generalizability. This separating hyperplane is called the optimal separating hyperplane (OSH). It is defined by *y* = *f*(***x***) = ***w***^T^***x***-*b* = 0, where ***w*** is the weight vector (*w*_1_,.., *w_d_*). The size of the margin is 2/||***w***||, so minimizing ||***w***||, the length of ***w***, maximizes the margin. SVM searches for the optimal decision function *y*(*x*), by minimizing ||***w***|| and requiring that *f*(***x****_i_*) < 0 if subject *i* has label *t_i_* = –1, and *f*(***x****_i_*) > 0 if *t_i_* = +1. Usually, however, we have overlapping class distributions and the two classes are not linearly separable. To solve this problem, subjects will be allowed to lie on the wrong side of the OSH, but with a penalty depending on their distance to the OSH. Slack variables ξ are introduced to quantify this so-called soft margin: A correctly classified subject has ξ = 0, otherwise ξ is the distance between the OSH and the subject’s feature vector ***x***. Apart from maximizing the margin, the classifier now also tries to limit the number of training errors, by penalizing non-zero ξs. A tunable parameter *C* controls the trade-off between margin and penalty, or equivalently, the choice between a more complex model with fewer training errors, or more training errors in a less complex, i.e., better generalizable, model. It was shown earlier (Franke et al., 2010) that tuning *C* can increase the model’s performance. We used the Matlab toolbox LIBSVM to perform SVM classification.

#### Step 2

The resulting classification model was tested through a leave-one-out cross-validation (LOOCV). Although LOOCV tends to have higher variance than *k*-fold CV (with, e.g., *k* = 5 or 10), the latter is prone to bias. Given the relatively small sample size (per age group), this bias might be relatively large, which led us to use the LOO-CV scheme. In LOOCV, the SVM model was trained using all participants except one, after which this model was used to predict the label of the left-out participant. The same procedure was carried out subsequently leaving out each participant once. The predicted labels of the participants were compared to the true labels to evaluate the accuracy of the model, which was assessed by three quantities: Sensitivity = TP/(TP+ FN), where TP is the number of true positives (correctly classified FR), and FN is it the number of false negatives. Specificity = TN/(TN+ FP), where TN is the number of true negatives (correctly classified TD), and FP is the number of false positives. The average, or balanced, accuracy = (sensitivity + specificity)/2. We also performed a receiver operating characteristic (ROC) analysis to assess the classifier’s performance for various settings of the discrimination threshold. We report the area under the curve (AUC).

For each model, a permutation procedure was carried out to test the significance of the prediction accuracy (Golland and Fischl, 2003). For each input dataset, we randomly permutated the labels of the subjects 1000 times, and a model was built from each permutated data set. For each of these models, prediction accuracy was calculated using the LOOCV procedure as described above. This yields a distribution of accuracies found from randomly labeled data. The accuracy of our true model was tested against this null distribution: the *p*-value was calculated as *N_h_*/*N_p_*, with *N_h_* the number of permutation accuracies higher than the true accuracy, and *N_p_* the number of permutations (1000).

#### The Prediction Models

Because vocabulary size and group-level differences therein varied across ages, we trained SVM for each age separately. First, we used the composite scores as features and trained the SVM with REC and PRO features separately. For both feature sets, each participant was represented by a feature vector containing the scores of the four composites. Since SVM can easily deal with larger amounts of features and discover the characteristics in the combination of features across composites, our next step was to train models using the scores of individual categories as features. Each participant was now represented by a vector containing 22 features, either REC or PRO. SVM’s computational performance has been found to be optimal when the feature values lie between -1 and 1, so we scaled the features by dividing them by the maximum attained score within each age group.

#### Results and Discussion

The performance of the models for each age is given in Table A1 of the Appendix. Only the 18-month PRO model yielded an accuracy (61%) significantly above chance level with a sensitivity of 63% and a specificity of 58%. Although significant group-level differences were found for PRO at 20 months, the accuracy of the individual prediction based on these features was not significant at this age. The reason that the composite class scores did not yield high prediction accuracy was probably due to the variation contained in each composite. The FR children might know many words in one category and relatively few in another as compared to TDs, although both categories belong to the same composite. Such opposite effects may cancel each other out and render the composite score uninformative.

**FIGURE 4:**
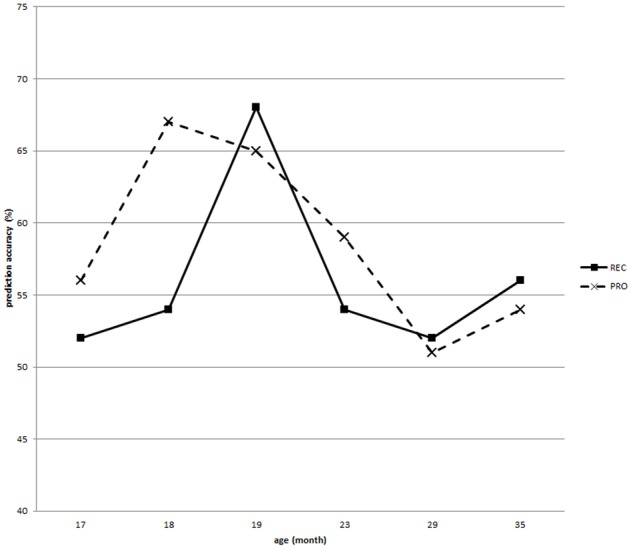
**Performance of the FR/TD prediction models trained on scores of the 22 individual N-CDI categories**. REC means receptive score and PRO means productive score.

The performance of the models based on the scores of each of the 22 individual categories is shown in **Table 10** and **Figure 4**, as a function of age.

**Table 10:** Performance of the FR/TD prediction models trained on receptive (REC) and productive (PRO) scores of the 22 individual N-CDI categories; ^∗^indicates significant accuracy.

Age		Accuracy	Sensitivity (%)	Specificity (%)	AUC
17 m	REC	52%	54	51	0.54
	PRO	56%	63	49	0.57
18 m	REC	54%	63	46	0.55
	PRO	**67%^∗^, *p* < 0.01**	65	68	0.68
19–20 m	REC	**68%^∗^, *p* < 0.001**	65	72	0.71
	PRO	**65%^∗^, *p* < 0.01**	67	63	0.66
23 m	REC	54%	50	58	0.54
	PRO	**59%^∗^, *p* < 0.01**	63	54	0.56
29 m	REC	52%	54	50	0.56
	PRO	51%	55	47	0.52
35 m	REC	56%	15	96	0.49
	PRO	54%	20	88	0.51

*Note that the high sensitivity at 35 m is meaningless: The overall significance is very low and not significant; AUC = 0.49–0.51, reflecting no possible discrimination. The actual balance between sensitivity and specificity is more or less random in this case*.

Consistent with the statistical analyses, the highest prediction accuracy was reached at 20 months (68%) when the model was trained with receptive scores of each individual category, with a fairly balanced sensitivity (65%) and specificity (72%). Interestingly, as shown in **Figure 4**, it seems that the accuracies for productive measures peaked earlier than receptive ones. Productive vocabularies before 18 months are very small, so even a tiny difference may have quite a dramatic impact.

**FIGURE 5:**
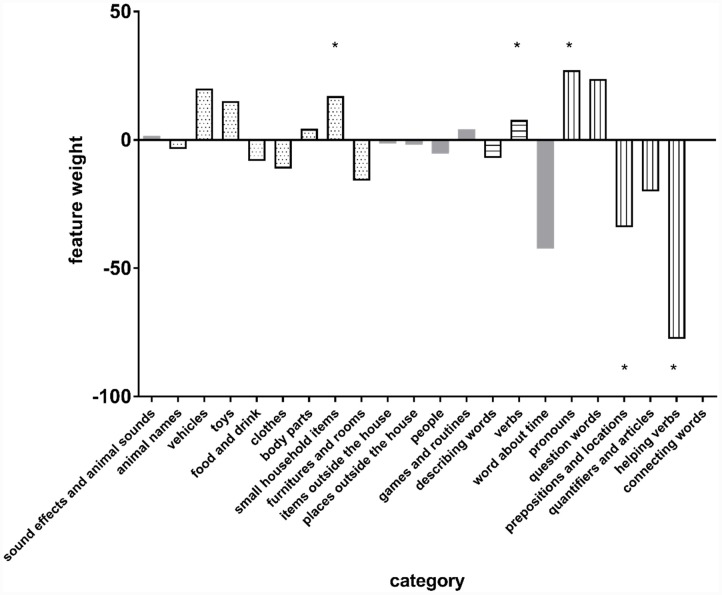
**Feature weights of the FR/TD prediction model trained with the 20-month-old productive (PRO) vocabulary of each individual category**. Dotted bars represent common noun categories; bars with horizontal stripes represent predicates categories; bars with vertical stripes represent closed class categories; gray bars represent other categories.

The feature weights of the most accurate models (i.e., the 18-month-old PRO, 20-month-old REC and PRO, and 23-month-old PRO) are listed in Table A2 in the Appendix. The weights of the model trained with 20-month-old productive scores are shown in **Figure 5**. Significance of the weights was tested with the permutation procedure as described above. The sign of a weight reflects whether an increase or decrease in the feature’s value contributes to being classified as FR, while its magnitude indicates how important the feature is for the classification of the two groups.

As can be seen from **Figure 5**, producing fewer words in the categories “prepositions and locations” and “helping verbs” makes an infant at age 20 months more likely to be classified as FR. It should be noted, however, that the features do not ‘act on their own’, but contribute to the classification in an interactive way. Producing fewer words in one category should thus be viewed in relation to the numbers of words produced in the other 21 categories. For instance, the weight of “verbs” production is significantly positive. In combination with, e.g., the negative weight of “helping verbs”, this means that when two children produce the same number of words at age 20 months, the FR child produces more verbs and fewer helping verbs than the TD child. At group level, however, the FR children produce fewer words in both categories; this is because the FR children’s vocabulary development as a whole is lagging behind as compared to that of the TD children.

With regard to the closed class words, although they contributed significantly to the prediction at 20 months, the individual categories can have opposite weights. Such opposite effects might be due to the heterogeneity of the words. Pronouns such as “I” and “you” have a simple phonological structure and tend to be prominent in speech, which might be beneficial for FR children. Helping verbs are abstract and relatively inconspicuous in running speech, which may make them difficult for FR to learn. The weights of the individual closed class categories suggest that in FR children’s composite closed class vocabulary, pronouns may be relatively well developed, whereas helping verbs and prepositions are underdeveloped. If so, the profile of early closed-class words production may be a marker for later language difficulties. As stated above, 20-month-old children are at the onset of quick vocabulary expansion, and it seems that at this early stage, some of the closed-class words are particularly difficult for FRs. Such words seem to be those that are abstract in meaning and not-so-prominent in running speech. Taking the FRs’ sustained difficulty with the closed class words in the group level analysis into consideration, it would be interesting for future studies to investigate whether FRs’ poor performance on closed class words as a whole is mainly driven by their difficulties with certain subclasses of the closed class words, and if so, whether knowledge of these subparts can be an early marker of reading difficulty. At later ages, closed-class words are not sufficient for individual classification, which suggests that FR children may have developed different strategies to learn these words, and as a group, they become more heterogeneous in terms of knowledge of the closed-class words.

The features that significantly contribute to the classification change between 18 and 19–20 months (Table A2 in Appendix). The distinctive features also differ for receptive and for productive vocabulary. This suggests a highly dynamic development of children’s vocabulary in this age range. Interestingly, within a composite, features can have weights with different signs. The opposing weights of features within one composite category suggest that the composites do not adequately reflect the individual children’s vocabulary profile.

We also examined the correlation between group-level difference and corresponding feature weights for each model, respectively. The effect sizes (Cohen’s *d*) for the TD-FR differences of individual N-CDI categories and the weights of the corresponding features correlated significantly for all significant models (Table A3 in the Appendix). This indicates that the feature weights used to predict at-risk status at individual level are consistent with group-level characteristics.

The machine learning method works in a bottom-up fashion, and it discovers patterns in the data without predetermined factors. It allows us to train models with a large amount of features, whose functions cannot be hypothesized a priori. As can be seen, the model works better when trained with individual categories in N-CDI than with the composites, although the latter have been assumed to have a stronger theoretical basis. Machine learning makes use of the multi-dimensionality of the input data, and is able to capture the subtle differences between groups that cannot be specified beforehand. Vocabulary is only one aspect in language development, and the predicting model can further improve when it is combined with more diversified measurements of other language abilities.

## Conclusion

In this study we investigated the trajectory of early vocabulary development of FR and TD toddlers, and we trained machine learning algorithms to predict whether an individual child is FR or TD. TD and FR children’s vocabulary was examined at 17, 18, 19–20, 23, 29, and 35 months using the Dutch version CDI. We found that there is a specific age period, 19-20 months, in which both total vocabulary and vocabulary composition are different for at risk and control children. Our results also suggest that closed-class words may be particularly difficult for FR children, where the FR children seem to consistently lag behind.

Importantly, we demonstrate that it is possible to train an SVM algorithm to predict the status of at risk based on their N-CDI scores with 68% accuracy. To predict the FR status, each individual child was classified by the SVM. Crucially, the child whose risk was to be predicted was not part of the sample from which the prediction model is constructed. Our findings indicate that the machine learning method may be fruitfully employed for early prediction of dyslexia. Consistent with the group level analyses, there is a specific age period, 19–20 months, in which the model is sensitive to predict the status of being at risk. At this age, the machine learning model also indicated that knowing fewer words in the “helping verbs” and “prepositions and locations” is a significant marker for being at family risk.

It should be acknowledged that we did not predict the manifestation of dyslexia, but only elevated risk. The children tested in this study are not old enough to know their final reading status. Hence the results of the current study cannot be used for early screening purposes yet. We will follow these children up, and the ultimate goal is to apply SVM to discriminate between the FR children who develop dyslexia and who do not at an early age when the reading status of these children is known. Individual prediction will help to establish the link between early vocabulary development, familial risk, and dyslexia.

## Author Contributions

AC, FW, CK, and HS contributed to the design of the study and to the analysis and interpretation of the data. FW and CK collected the data. AC, FW, CK, and HS contributed to the drafting and revising of the manuscript.

## Conflict of Interest Statement

The authors declare that the research was conducted in the absence of any commercial or financial relationships that could be construed as a potential conflict of interest.

## Footnotes

1 Besides one parent, DDP also requires one first-degree family member to be reading impaired for an infant to be FR.

2 We did not analyze “other” as a composite in Study I since the categories in “other” are heterogeneous. However, in order to make full use of the data for individual prediction, “other” was used in machine learning.
