# Supplementary material for: Individualized Early Prediction of Familial Risk of Dyslexia: A Study of Infant Vocabulary Development
Source: Front Psychol. 2017 Feb 21;8:156. doi: 10.3389/fpsyg.2017.00156 (PMC5318442; doi:10.3389/fpsyg.2017.00156)
Supplement: Supplementary file 1 [file Data_Sheet_1.docx]

**Appendix**

Table A1. Model performance for each age group, taking composite scores as features. REC indicates models trained with receptive scores, and PRO indicates models trained with productive scores. * indicates the model that yielded a significant accuracy.

|  | accuracy | sensitivity | specificity |
| --- | --- | --- | --- |
| 17m REC | 48% | 26% | 70% |
| 17m PRO | 50% | 82% | 19% |
| 18m REC | 55% | 67% | 44% |
| 18m PRO* | 61% | 63% | 58% |
| 20m REC | 52% | 48% | 55% |
| 20m PRO | 58% | 80% | 37% |
| 23m REC | 46% | 37% | 56% |
| 23m PRO | 56% | 63% | 49% |
| 29m REC | 55% | 44% | 66% |
| 29m PRO | 50% | 49% | 52% |
| 35m REC | 54% | 18% | 88% |
| 35m PRO | 53% | 35% | 71% |

Table A2. Feature weights of the most accurate models trained with scores of individual categories. * indicates the significant features. PRO = productive, and REC = receptive.

|  | 18m PRO | p value | 20m REC | p value | 20m PRO | p value | 23m PRO | p value |
| --- | --- | --- | --- | --- | --- | --- | --- | --- |
| Sound effects | 0 | 1 | 5.19 | 0.49 | 1.54 | 0.78 | 0.61 | 0.54 |
| Animal names | -1.63 | 0.46 | -6.77 | 0.09 | -2.74 | 0.57 | -3.06 | 0.07 |
| Vehicles | 0.34 | 0.91 | -7.69 | 0.36 | 20.00 | 0.12 | -0.14 | 0.85 |
| Toys | -4.48 | 0.36 | -5.38 | 0.53 | 15.09 | 0.24 | 0.63 | 0.38 |
| Food and drink | -3.03 | 0.29 | -4.11 | 0.36 | -7.43 | 0.15 | -0.19 | 0.88 |
| Clothes | -1.80 | 0.63 | 2.71 | 0.74 | -10.38 | 0.35 | 1.20 | 0.25 |
| Body parts | -4.96 | 0.15 | 1.24 | 0.85 | 4.33 | 0.49 | -0.35 | 0.80 |
| Small household items | 0.68 | 0.85 | 2.00 | 0.75 | 17.08 | 0.05 | 0.73 | 0.59 |
| Furniture and rooms | 2.64 | 0.57 | 10.58 | 0.20 | -15.14 | 0.17 | 0.38 | 0.67 |
| Items outside the house | -9.82 | 0.06 | 1.53 | 0.83 | -0.68 | 0.94 | -0.53 | 0.63 |
| Places outside the house | -0.07 | 0.99 | -1.61 | 0.87 | -1.2 | 0.95 | -0.63 | 0.28 |
| People | 12.22* | 0.01 | 0.81 | 0.93 | -4.61 | 0.64 | 0.66 | 0.42 |
| Games and routines | 5.50 | 0.06 | 13.05 | 0.11 | 4.17 | 0.48 | 0.65 | 0.50 |
| Descriptive words | 5.94 | 0.13 | -6.03 | 0.14 | -6.35 | 0.40 | -0.99 | 0.42 |
| Verbs | 1.24 | 0.66 | 3.10 | 0.27 | 7.81* | 0.05 | -1.39 | 0.44 |
| Words about time | -2.84 | 0.44 | -13.17 | 0.16 | -41.58 | 0.21 | 0.90 | 0.10 |
| Pronouns | -0.34 | 0.96 | 2.64 | 0.69 | 27.16* | 0.03 | -0.10 | 0.92 |
| Question words | 0 | 0.66 | 17.09 | 0.11 | 23.78 | 0.67 | -0.06 | 0.86 |
| Prepositions and locations | 1.55 | 0.72 | -14.32* | 0.03 | -33.27* | 0.00 | -0.84 | 0.49 |
| Quantifiers and articles | -5.06 | 0.29 | -9.30 | 0.32 | -19.32 | 0.32 | -1.03 | 0.09 |
| Helping verbs | 0.73 | 0.84 | 3.61 | 0.63 | -76.74* | 0.03 | 0.41 | 0.47 |
| Connecting words | 0 | 1 | -5.62 | 0.549 | 0 | 0.94 | 0.09 | 0.533 |

Table A3. Correlation between FR/TD group-level differences and corresponding feature weights of the models trained with individual N-CDI categories that yielded highest prediction accuracy. REC indicates models trained with receptive scores and PRO indicates models trained with productive scores.

| Model | Pearson’s r | Significance (2-tailed) |
| --- | --- | --- |
| 18m PRO | 0.78 | p<0.001 |
| 20m REC | 0.92 | p<0.001 |
| 20m PRO | 0.71 | p<0.001 |
| 23m PRO | 0.79 | p<0.001 |
